# RIP kinases and necroptosis in aging and aging-related diseases

**DOI:** 10.1093/lifemedi/lnac003

**Published:** 2022-06-14

**Authors:** Yuanxin Yang, Xingyan Li, Tao Zhang, Daichao Xu

**Affiliations:** Interdisciplinary Research Center on Biology and Chemistry, Shanghai Institute of Organic Chemistry, Chinese Academy of Sciences, Shanghai 201210, China; University of Chinese Academy of Sciences, Beijing 100049, China; Interdisciplinary Research Center on Biology and Chemistry, Shanghai Institute of Organic Chemistry, Chinese Academy of Sciences, Shanghai 201210, China; Department of Pathology, Beth Israel Deaconess Medical Center, Harvard Medical School, Boston, MA 02215, United States; Interdisciplinary Research Center on Biology and Chemistry, Shanghai Institute of Organic Chemistry, Chinese Academy of Sciences, Shanghai 201210, China

**Keywords:** RIPK1, RIPK3, necroptosis, aging, inflammation

## Abstract

Aging is a natural process that is characterized by chronic, low-grade inflammation, which represents the primary risk factor in the pathogenesis of a variety of diseases, i.e. aging-related diseases. RIP kinases, in particular RIPK1 and RIPK3, have emerged as master regulators of proinflammatory responses that act either by causing apoptosis and necroptosis or by directly regulating intracellular inflammatory signaling. While, RIPK1/3 and necroptosis are intimately linked to multiple human diseases, the relationship among RIPK1/3, necroptosis, and aging remains unclear. In this review, we discuss current evidence arguing for the involvement of RIPK1/3 and necroptosis in the progression of aging. In addition, we provide updated information and knowledge on the role of RIPK1/3 and necroptosis in aging-related diseases. Leveraging these new mechanistic insights in aging, we postulate how our improved understanding of RIPK1/3 and necroptosis in aging may support the development of therapeutics targeting RIPK1/3 and necroptosis for the modulation of aging and treatment of aging-related diseases.

## Introduction

Aging is an irreversible and inevitable biological process that is characterized by time-dependent cellular and functional decline, resulting in deterioration of physical and mental conditions of the individual organism [1]. In line with this, aging is one of the main risk factors in the occurrence of many aging-related disorders, including neurodegenerative diseases, cardiovascular diseases, cancer, and other diseases [2]. Collectively, aging-related diseases represent a formidable global financial burden and a significant healthcare challenge. Thus, identifying effective preventive approaches and therapeutic methods that halt the progression of multiple aging-related pathological conditions are urgently needed.

Among the many tentative hallmarks of aging, inflammaging, which is characterized by chronic, low-grade sterile inflammation that occurs with age, has emerged as a key feature of aging across diverse species [3]. Inflammaging plays an important role in the underlying mechanisms of both physiological and pathological aging and aging-related diseases [3]. One of the common features of inflammaging is the aging-related increase in the level of proinflammatory cytokines including tumor necrosis factor α (TNFα), interleukin 1β (IL1β), and IL6 [4]. Increased levels of TNFα, IL1β, and IL6 in the serum of elderly are associated with the development of several aging-related diseases, including Alzheimer’s disease (AD) [5] and type 2 diabetes [6].

RIPK1 and RIPK3 are members of RIP (receptor-interacting protein) family of protein serine/threonine kinases. RIPK1 has emerged as a central mediator of both cell survival and cell death. The activation of RIPK1 can dictate cell survival independent of its kinase activity or promote cell death and inflammation mediated by its kinase activity [7]. RIPK1 is composed of an amino-terminal kinase domain (KD), a carboxy-terminal death domain (DD) and a bridging intermediate domain (ID) with a RIP homotypic interaction motif (RHIM) [8]. The KD of RIPK1 is important in initiating multiple cell death pathways, including apoptosis, necroptosis, and pyroptosis [7], which has become an important drug target for small molecular inhibitors, the prototype of which is necrostatin-1s (Nec-1s) [9]. RIPK3 bears an amino-terminal KD and a carboxy-terminal region that harbors a RHIM [10, 11]. RIPK3 is a downstream mediator of RIPK1 via their respective RHIM interaction in driving necroptosis [12].

Necroptosis is an inflammatory cell death pathway that has emerged as an important pathway in driving the pathology of human diseases, including multiple aging-related diseases [13]. Necroptosis can be activated by RIPK1 kinase, which in turn promotes the activation of RIPK3 or activated by the interaction of RIPK3 with other RHIM-containing proteins such as TRIF or ZBP1 [14]. Activation of RIPK3 is a key step in initiating necroptosis, which in turn phosphorylates MLKL to induce a conformational change, oligomerization, and translocation to the plasma membrane to promote cell lysis [15, 16]. Cells undergoing necroptosis are characterized by cell swelling, loss of plasma membrane permeability, membrane rupture, the release of damage-associated molecular patterns (DAMPs), and increased production of proinflammatory cytokines, such as TNFα, IL1β, and IL6 [14, 17]. Numerous studies have shown that use of genetic and pharmacological manipulations that inhibit necroptosis can reduce inflammation induced in a variety of systems [17, 18].

Due to the importance of necroptosis in driving inflammation and the pathology of degenerative diseases, recent advances have proposed that inflammaging and aging-related tissue degeneration could be a result of an aging-related increase in necroptosis (Table 1). In this review, we will examine the relationship among RIPK1/3, necroptosis, aging and disease and propose novel molecular links between necroptosis pathway and the hallmarks of aging, as well as possible implications for antiaging therapeutic interventions.

**Table 1.** Activation of RIPK1/3 and necroptosis in aging and aging-related diseases

Aging and aging-related diseases	Activation of RIPK1	Activation of RIPK3	Activation of MLKL	Amelioration/Rescue
Brain aging	RIPK1↑ [79]	RIPK3↑ [79]	MLKL↑ [79], p-MLKL↑ [79, 80] oligomerized-MLKL↑ [79]	*Mlkl*-KO [79, 80], RIPK1 inhibitor Nec-1 s [79]; RIPK3 inhibitor GSK872 [80]
Liver aging	p-RIPK1 [81]	RIPK3 and p-RIPK3↑ [81, 83]	MLKL and p-MLKL↑ [81, 83]	Nec-1s [81, 83]
Inflammaging	RIPK1↑ [74]	p-RIPK3↑ [74]	MLKL and p-MLKL↑ [74, 71]	CR [74]
Testis aging	ND	ND	p-MLKL↑ [84]	*Ripk3*-KO, *Mlkl*-KO and RIPK1 inhibitor RIPA-56 [84]
Cochlea aging	RIPK1↑ [88]	RIPK3↑ [88]	MLKL↑ [88]	
AD	RIPK1↑ [90, 92, 93]; p-RIPK1↑ [91, 93]	p-RIPK3↑ [91]	MLKL↑ p-MLKL↑ [90–92]	Nec-1s [90, 93]; *Mlkl*-KO and GSK872 [92]; *Ripk1*-D138N [93]
PD	RIPK1↑ [96, 97]	RIPK3↑ [96, 97]	MLKL↑ [96, 97]; p-MLKL↑ [98]	*Ripk3*-KO and *Mlkl*-KO [96–98]; Nec-1s [98]
ALS	RIPK1↑ [102, 103]; p-RIPK1↑ [102]	RIPK3↑ [102]	MLKL and p-MLKL↑ [102]	*Ripk3*-KO, *Ripk1*-D138N, and Nec-1s [102]
Cancer	RIPK1↑ [111–114]	RIPK3↓ [104–108], RIPK3↑ [120, 121]	MLKL↓ [104–108], MLKL↑ [121]	Nec-1s [115, 123], RIPK1 inhibitor GSK963 [116]; *Ripk3*-KO [123]
Osteoarthritis	RIPK1↑ [127]	RIPK3↑ [127–129]	MLKL↑ [128, 129]	Nec-1s [126–129]
Myocardial ischemia-reperfusion (I/R) injury	RIPK1↑ [132]	RIPK3↑ [132, 135]	MLKL↑ [132]	Nec-1 [131, 132]; *Ripk3*-KO [133–135]
Diabetes mellitus	ND	RIPK3↑ [138, 141]	MLKL↑ [139]	*Mlkl*-KO and Nec-1s [139]
Atherosclerosis	RIPK1↑ [147]	RIPK3↑ [144, 145]	MLKL↑ [144, 145]	*Ripk3*-KO [145, 146]; Nec-1s [144]; RIPK1 inhibitor GSK-547 [148]
Age-related macular degeneration	ND	RIPK3 aggregation↑ [151, 153]	ND	Nec-1s [151], *Ripk3*-KO [154]
Nonalcoholic fatty liver disease	RIPK1↑ [162]	RIPK3↑ [158, 159]	MLKL↑ [162]	*Ripk3*-KO [158, 159]; RIPK1 inhibitor RIPA-56 [162]
Retinal degeneration	RIPK1↑ [167]	RIPK3↑ [167]		Nec-1s [167]
**CKD**	RIPK1↑ [172, 174]	RIPK3↑ [172–174]	MLKL↑ [172]	Nec-1s [170–174]; *Ripk3*-KO [175]
COVID-19	RIPK1↑ [184]; p-RIPK1↑ [187]	RIPK3↑ [184]	p-MLKL↑ [183]; MLKL↑ [184]	Nec-1s [187]

Abbreviations: KO, knockout; ND, not determined.

## The diverse functions of RIPK1

RIPK1 acts downstream of many receptors, including death receptors, such as tumor necrosis factor receptor 1 (TNFR1), Fas, death receptors (DRs) 4 and 5, and Toll-like receptors, such as TLR3 and TLR4. Among these receptors, TNFR1 is the most extensively characterized receptor that mediates the diverse functions of RIPK1 [19, 20]. In addition, activation of RIPK1 kinase mediated by TNFR1 has been show to promote most of the deleterious effects activated by TNFα in human diseases [13]. Current genetic and mechanistic insights indicate that RIPK1 plays pivotal roles in two branches of the TNFR1 response: the scaffold function inhibits apoptosis and necroptosis via NF-κB-mediated production of antiapoptotic genes, and restraining ZBP1 and TRIF-mediated RIPK3 activation, respectively; the kinase function promotes apoptosis, necroptosis and induces inflammation [7]. These diverse functions of RIPK1 are precisely controlled by different cellular contents, as described below.

### TNFR1-mediated RIPK1 signaling

**Figure 1.**
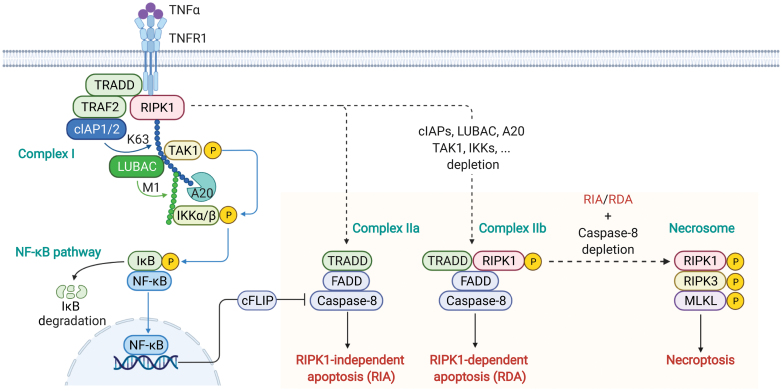
Activation of TNFR1 mediates NF-κB pathway activation, RIPK1-independent apoptosis, RIPK1-dependent apoptosis and necroptosis. Upon TNFα stimulation, TNFR1 is trimerized to form complex I in associated with the intracellular DD of TNFR1. The adaptor protein TRADD and RIPK1 both contain a DD and are recruited first to the TNFR1. TRADD then recruits TRAF2 and cIAP1/2 to perform K63-linked ubiquitination of RIPK1, which acts as a scaffold to recruit LUBAC complex composed of HOIP, HOIL1 and SHARPIN. LUBAC performs M1-linked ubiquitination of RIPK1. The K63 and M1-linked ubiquitin chains recruit TAK1 and IKKα/IKKβ to complex I, respectively. The M1 ubiquitin chains also recruit A20 to complex I, which is a suppressor of RIPK1 activity and NF-κB activation by deubiquitinating K63-linked ubiquitin chains on RIPK1. The kinases, including TAK1, and IKKα/β suppress the activation of RIPK1 by direct inhibitory phosphorylation. When the pro-survival mechanisms emanating from complex I are compromised, TNFR1 can induce apoptosis by RIPK1-independent (complex IIa) or RIPK1-dependent (complex IIb) activation of FADD/caspase-8. When caspases are inhibited by zVAD.fmk, activated RIPK1 binds to RIPK3 to form necrosome, which results in MLKL activation and necroptosis. Abbreviations: TNFR1, TNF receptor 1; TRADD, TNFR1-associated death domain protein; RIPK1/3, receptor-interacting protein kinase 1/3; TRAF2, TNF receptor-associated factor 2; cIAP1/2, cellular inhibitors of apoptosis 1 and 2; LUBAC, linear ubiquitin chain assembly complex; TAK1, transforming growth factor β-activated kinase 1; IKKα/β, IκB kinase α/β; IκB, inhibitor of NF-κB; NF-κB, nuclear factor-κB; FADD, Fas-associated protein with death domain; MLKL, mixed lineage kinase like.

Upon TNFα ligation, RIPK1 is recruited to TNFR1 together with TRADD to form a transient membrane signaling complex named TNFR1 signaling complex (TNF-RSC) or complex I^21^ (Fig. 1). TRADD and RIPK1 are the first two components recruited to complex I mediated by homotypic interaction via the binding to intracellular DD of trimerized TNFR1 with their own DDs [21]. In complex I, TRADD recruits the E3 ubiquitin ligases cIAP1 and cIAP2 via the adaptor TRAF2 [22], which in turn performs K63-linked ubiquitination of RIPK1 in complex I [23]. The linear ubiquitination assembly complex (LUBAC), which is recruited by binding with K63-linked ubiquitin chains in complex I, performs M1-linked ubiquitination of RIPK1 [24–26]. The K63-linked ubiquitin chains on RIPK1 allow the recruitment of TAK1/TAB2/TAB3 complex [23], while the M1-linked ubiquitin chains on RIPK1 facilitates the recruitment of NEMO/IKKα/IKKβ complex [24]. Subsequently, activated TAK1 phosphorylates and activates IKKα/IKKβ, which mediates canonical NF-κB signaling by phosphorylating IκB, thereby inducing its proteasomal degradation [27]. Activated NF-κB pathway transcriptionally induces the expression of pro-survival genes, such as cellular FLICE-like inhibitory protein (cFLIP) [28], which encodes the catalytically inactive homolog of caspase-8 to prevent apoptosis [29]. Blocking the activation of NF-κB-mediated transcription (e.g. actinomycin D) or translation (e.g. cycloheximide) leads to the formation of a cytoplasmic, cell death-inducing complex named complex IIa [30] (Fig. 1). This RIPK1 kinase-independent complex IIa involves interaction between TRADD, Fas-associated protein with death domain (FADD) and caspase-8, which promotes the activation of caspase-8 to mediate apoptosis [30]. The complex I is the first checkpoint in TNFR1 signaling that decides whether the cells activate NF-κB and survive or die by apoptosis or necroptosis (Fig. 1).

### RIPK1 scaffold function prevents apoptosis and necroptosis

Mice with RIPK1 universal knockout are born at the expected Mendelian ratios but die perinatally [31]. Genetic studies on mice suggest that the perinatal lethality of *Ripk1*^−/−^ mice is caused by both apoptosis mediated by FADD/caspase-8 and necroptosis mediated by RIPK3/MLKL [31]. In line with this, *Ripk1*^−/−^*Fadd*^−/−^*Ripk3*^−/−^, *Ripk1*^−/−^*Casp8*^−/−^*Ripk3*^−/−^, and *Ripk1*^−/−^*Fadd*^−/−^*Mlkl*^−/−^ mice are fully rescued from perinatal lethality and live to adulthood [31]. While, loss of RIPK1 in mice leads to perinatal lethality, homozygous loss-of-function mutations of RIPK1, including frameshift, missense, and nonsense mutations in human individuals, cause severe immunodeficiency, inflammatory bowel diseases, and arthritis [32–34]. In addition, loss of RIPK1 in human individuals promotes the pediatric onset of primary immunodeficiency with chronic enteropathy [34].

**Figure 2.**
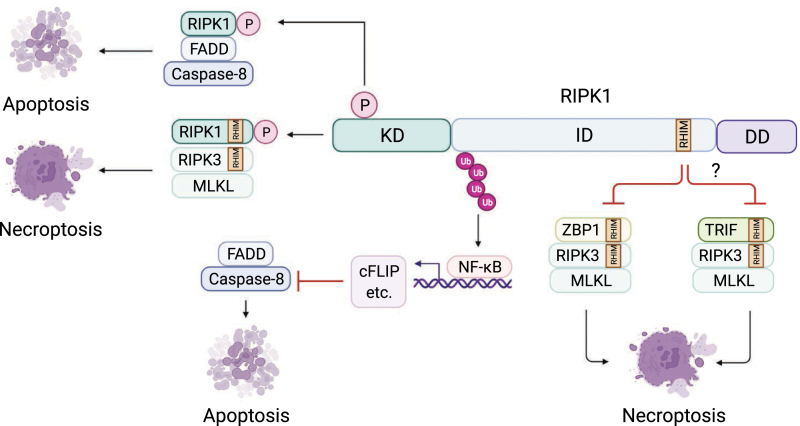
RIPK1 kinase function promotes apoptosis and necroptosis, while its scaffold function prevents apoptosis and necroptosis. Upon kinase activation, as measured by the autophosphorylation of RIPK1 S166, RIPK1 binds to FADD and caspase-8 to form complex IIb to promote the activation of caspases and execution of RIPK1-dependent apoptosis (RDA). In the setting that when caspase-8 is inhibited, activated RIPK1 recruits RIPK3 via its RHIM domain to form the necrosome for the execution of necroptosis. In the absence of kinase function, the scaffold function of RIPK1 inhibits apoptosis via NF-κB-mediated production of antiapoptotic genes, such as cFLIP, which inhibits caspase-8 activation. The scaffold function of RIPK1 inhibits necroptosis via its RHIM domain, which restrains ZBP1 and TRIF-mediated RIPK3 activation. Abbreviations: ZBP1, Z-DNA Binding Protein 1; TRIF, TIR-domain-containing adapter-inducing interferon-β; RHIM, RIP homotypic interaction motif.

As described above, RIPK1 has an important role in mediating NF-κB activation, which transcriptionally induces the expression of antiapoptotic genes, such as cFLIP, to inhibit caspase-8 activation and prevent apoptosis [28, 29]. This process is independent of RIPK1 kinase activity, as neither a kinase-dead RIPK1 mutant nor the pharmacological inhibition of RIPK1 kinase activity has any effect on the TNFα-stimulated activation of NF-κB [7]. These results suggests that mediating NF-κB activation by RIPK1 is an essential part of the RIPK1 pro-survival scaffold function by preventing apoptosis (Fig. 2).

The mechanism by which RIPK1 scaffold function prevents necroptosis was revealed recently. The RHIM in the ID of RIPK1 has been shown to prevent necroptosis. Mice with *Ripk1*^*RHIM/RHIM*^ mutation also die around birth due to massive necroptosis, but not caspase-8-dependent apoptosis [35]. *Ripk1*^*RHIM/RHIM*^ embryos at embryonic day 18.5 (E18.5) develop RIPK3/MLKL-dependent necroptosis in skin but lack the caspase-8-dependent intestinal apoptosis seen in *Ripk1*^−/−^ embryos, suggesting that mutation of the RIPK1-RHIM primarily unleashes the RIPK3/MLKL-dependent necroptosis pathway. Accordingly, *Ripk1*^*RHIM/RHIM*^*Ripk3*^−/−^ and *Ripk1*^*RHIM/RHIM*^*Mlkl*^−/−^ mice are viable and fertile [35]. Thus, the RHIM of RIPK1 is essential for RIPK1 scaffold function to preventing necroptosis during development. Mechanistic studies suggest that another two RHIM-containing proteins, ZBP1 and TRIF, are involved in mediating RIPK3/MLKL-dependent necroptosis in *Ripk1*^−/−^ mice. ZBP1 was identified as a sensor of Z-DNA and Z-RNA involved in the activation of innate immune responses [36], while TRIF is an adaptor protein downstream of TLR3 and TLR4, which are pathogen-recognition receptors of the Toll-like receptor family [37]. Combined knockout of ZBP1 and TRIF can inhibit necroptosis in *Ripk1*^−/−^ mice and rescue perinatal lethality in *Ripk1*^−/−^*Casp8*^−/−^ mice [35]. In addition, ZBP1 deficiency also protects *Ripk1*^*RHIM/RHIM*^ mice from perinatal lethality and inhibits RIPK3/MLKL-dependent necroptosis [35]. Taken together, these results suggests that ZBP1 and/or TRIF drive RIPK3/MLKL-mediated necroptosis when RIPK1 is absent (Fig. 2).

### RIPK1 kinase activity promotes apoptosis and necroptosis

While mice lacking RIPK1 die perinatally, mice expressing kinase-inactive mutants of RIPK1, such as RIPK1-K45A mutant or RIPK1-D138N mutant, are viable and highly resistant to TNFα-induced cell death and inflammatory conditions associated with chronic infection, sepsis, degenerative conditions, or other types of tissue injury [38–40]. Upon activation of the kinase, as measured by the well-established autophosphorylation of S166 [41, 42], RIPK1 can enact either apoptosis [RIPK1 kinase-dependent apoptosis (RDA)] or necroptosis (Fig. 2). In addition, RIPK1 kinase can promote inflammation in both cell death-dependent and independent manners [43, 44].

Complex I provides a critical checkpoint that decides if RIPK1 kinase is to be activated (Fig. 1). The activation of RIPK1 in complex I is determined by a code including complex ubiquitination and phosphorylation events on RIPK1, which include many known key regulators of NF-κB pathway, such as TAK1 complex [45], IKK complex [46], LUBAC complex [24], cIAPs [23], and A20 [47]. These proteins are also recruited into complex I to directly suppress the activation of RIPK1 by modulating the modification patterns on RIPK1 [7]. In human individuals, loss-of-function mutations in the genes that encode these key components of complex I lead to inflammatory and neurodegenerative diseases, which is largely stemmed from aberrant activation of RIPK1 [7].

The extent of RIPK1 kinase activity, which may be both cell type-specific and stimulus-specific, determines the mode of cell death. For example, in oligodendrocytes, TNFα stimulation alone may promote RIPK1 activation and cell death [41]; whereas in fibroblasts, sustained activation of RIPK1 kinase can only be achieved by stimulation with TNFα in combination with factors that reduce the inhibition of RIPK1, such as inhibition or depletion of cIAPs, TAK1, IKKs, or TBK1 [23, 45, 46, 48]. Activated RIPK1 then binds to TRADD, FADD, and caspase-8 to form complex IIb and promote activation of caspases and execution of RDA [20] (Fig. 1).

In experimental paradigms, necroptosis can be activated when either complex IIa or complex IIb is stabilized by caspases inhibitor, such as zVAD.fmk [9] (Fig. 1). While activated caspase-8 has been established as a critical mediator of apoptosis, it has emerged as a key suppressor of necroptosis. *Casp8*^*C362A/C362A*^ knock-in mice expressing catalytically inactive caspase-8 exhibit embryonic lethality due to unleashed necroptosis [49]. Knockout of RIPK3 in *Casp8*^*C362A/C362A*^*Ripk3*^−/−^ mice or MLKL in *Casp8*^*C362A/C362A*^*Mlkl*^−/−^ mice prevents the embryonic lethal phenotypes, establishing that the activity of caspase-8 suppresses necroptosis during embryonic development [49]. Activation of caspase-8 in complex II (including complex IIa and IIb) cleaves RIPK1 after residues D324 (D325 in mouse RIPK1), which inhibits interaction of RIPK1 and RIPK3 [49, 50]. Inhibition of caspase-8-mediated cleavage of RIPK1 by pan-caspases inhibitor (e.g. zVAD.fmk) leads to stabilization of complex II and subsequent accumulation of activated RIPK1, which is sufficient to recruit RIPK3 to form the necrosome (Fig. 1). In necrosome, RIPK3 is activated which in turn mediates the phosphorylation of MLKL to drive its activation and oligomerization for the execution of necroptosis. As summarized previously, these experimental manipulations mimic some aspects of human genetic deficiencies in various regulators of RIPK1 that lead to aberrant RIPK1 kinase activation and provide guidance for possible indications where RIPK1 inhibitors may be efficacious [13].

### RIPK1 kinase activity promotes inflammation

**Figure 3.**
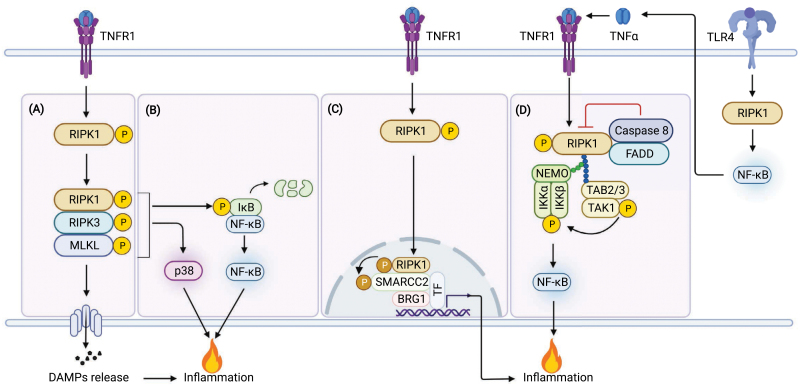
RIPK1 kinase activity drives inflammation in both cell death-dependent and independent manners. (A) RIPK1 kinase activity drives necroptosis, which promotes inflammation by the passive release of DAMPs from ruptured cell membrane. (B) The RIPK1 kinase-RIPK3-MLKL axis activates p38, and promotes the ubiquitination and degradation of IκBα that could activate NF-κB to mediate a prolonged inflammation during necroptosis. (C) In microglia-like BV2 cells, treatment with LPS induces autocrine production of TNFα, which promotes the activation of RIPK1 when caspase-8 or FADD is inhibited. Activated RIPK1 recruits TAK1 and IKK complexes to form a novel secondary signaling complex, which drives the activation of NF-κB pathway to induce a prolonged inflammation. (D) When RIPK1 is activated by extracellular stimuli, such as TNFα, activated RIPK1 translocates to nucleus to mediate the phosphorylation and activation of SMARCC2, a key component of the BAF complex, to drive chromatin remodeling and the transcription of proinflammatory genes. Abbreviations: SMARCC2, SWI/SNF related, matrix associated, actin dependent regulator of chromatin subfamily C member 2; BAF, BRG1/BRM-associated factor; BRG1, Brahma-related gene 1.

RIPK1 kinase activity can drive inflammation in both cell death-dependent and independent manners (Fig. 3). RIPK1 kinase activity is important for necroptosis, which is known to promote inflammation by the passive release of DAMPs from ruptured cell membrane [14]. In addition to DAMPs-induced inflammation, necroptosis can also promote inflammation cell-autonomously through intracellular signaling mechanisms including p38 and NF-κB pathway [51]. TNFα-induced necroptosis leads to two waves of cytokine production. The first wave is more transient and weaker than the second mediated by TNFα alone, which does not require the kinase activity of RIPK1; whereas the second wave depends on the kinase activity of RIPK1 and the necroptotic machinery, including RIPK3 and MLKL. Mechanistically, the RIPK1 kinase-RIPK3-MLKL axis activates p38, and promotes the ubiquitination and degradation of IκBα that could activate NF-κB [51] (Fig. 3). However, the mechanism by which RIPK1 kinase-RIPK3-MLKL axis activates p38 and promotes the ubiquitination and degradation of IκBα remains unknown.

When caspase-8 or FADD is inhibited, treatment with LPS induces two waves of proinflammatory cytokine production without inducing cell death in microglia-like BV2 cells [43]. The first wave is mediated by an early RIPK1 scaffold-dependent but RIPK1 kinase-independent mechanism; whereas the second wave requires RIPK1 kinase activity as well as its scaffold function to recruit TAK1 and IKK complexes to form a novel secondary signaling complex, which drives the activation of NF-κB pathway to induce inflammation [43]. Mechanistically, the activation of RIPK1 and the formation of the secondary signaling complex is mediated by autocrine production of TNFα [43] (Fig. 3). Thus, the kinase activity of RIPK1 can drive its scaffold function to promote inflammation in a novel NF-κB pathway, which is negatively regulated by caspase-8 and FADD.

A more recent study discovered that RIPK1 kinase activity can promote chromatin remodeling to mediate inflammation independent of cell death [44]. This study revealed a nuclear RIPK1/BAF complex that is involved in promoting the transcriptional induction of inflammation [44]. Mechanistically, nuclear RIPK1 is physically associated with the BAF complex, which is recruited by specific transcription factors to active enhancers and promoters marked by H3K4me1 and H3K27ac when RIPK1 is activated by extracellular stimuli, such as TNFα. Activated nuclear RIPK1 then mediates the phosphorylation and activation of SMARCC2, a key component of the BAF complex, to drive chromatin remodeling and the transcription of proinflammatory genes [44] (Fig. 3). Therefore, RIPK1 kinase activity serves a unique signaling function in the nucleus by transmitting signals from extracellular stimuli to the BAF complex to promote chromatin remodeling and transcription of proinflammatory factors.

## RIPK3 and the necroptosis signaling pathway

**Figure 4.**
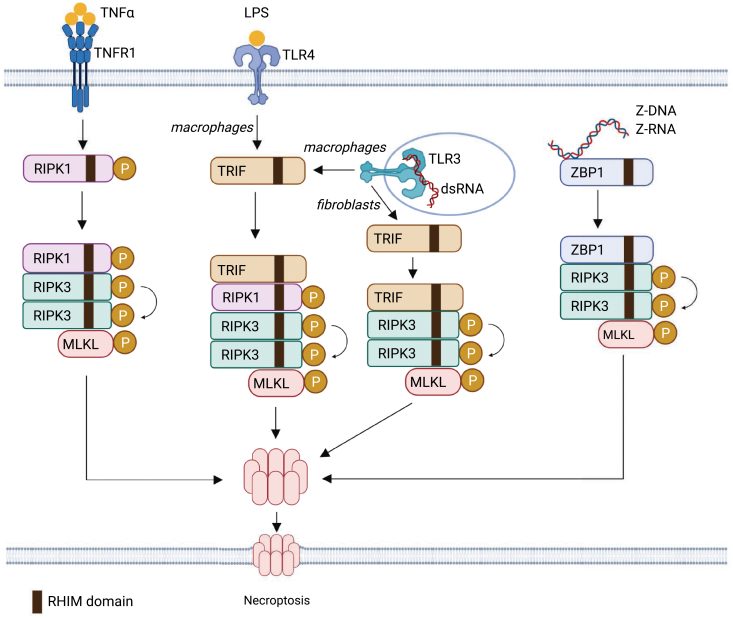
Regulation of necroptosis by RHIM-containing proteins. RIPK3 acts as a signal integrating molecule for necroptosis by receiving signals from diverse pathways via a homotypic RHIM interaction involving RIPK1, TRIF, and ZBP1. In receptor signaling pathways, necroptosis can be activated by the stimulation of TNFR1, TLR3 or TLR4 by TNFα, dsRNA or LPS, respectively, in conditions when caspase-8 of FADD is inhibited or depleted. The RHIM–RHIM interaction between RIPK1 and RIPK3 lead to RIPK3 activation. Activated RIPK3 in turn phosphorylates MLKL, leading to the oligomerization of MLKL, which is recruited to membranes, leading to membrane permeabilization and cell lysis. In addition to receptor signaling, necroptosis can be triggered by Z-DNA or Z-RNA-mediated ZBP1 activation, which instructs the assembly of cell death signaling scaffolds and facilitates RIPK3 activation via RHIM-induced interaction of RIPK3 and ZBP1.

The core of the necroptotic pathway involves RHIM-containing proteins, the activity of RIPK3, and the phosphorylation-driven activation by RIPK3 of MLKL (Fig. 4). There are four RHIM-containing proteins have been described in the mammalian proteome. In addition to RIPK1 and RIPK3, the adapter protein TRIF contains a RHIM [52], and the innate immune sensor ZBP1 contains three putative RHIMs [53], however, there is no more experiments to prove the existence and function of the third RHIM domain within ZBP1. The relationship between these four RHIM-containing proteins is complicated and context-dependent. In unstimulated condition, RIPK1 RHIM acts as a scaffold to prevent the RHIM–RHIM interaction of ZBP1 and RIPK3 to limit necroptosis [35] (Fig. 2). In line with this, ZBP1 deficiency alone can inhibit necroptosis in RIPK1 RHIM mutant mice and rescued perinatal lethality in the *Ripk1*^*RHIM/RHIM*^ mice [35]. Whereas in receptor signaling pathways, notably those initiated by TNFR1 and TLR3/4, which activate RIPK1, the RHIM of RIPK1 interacts with RHIM of RIPK3 to induce necroptosis [54, 55]. Therefore, RIPK3 can act as a signal integrating molecule for necroptosis by receiving signals from diverse pathways via a homotypic RHIM interaction involving RIPK1, TRIF, and ZBP1 (Fig. 4). We briefly consider each of these signaling pathways, and discuss specific physiological or pathological settings in which they can be activated as below.

The RHIM–RHIM interaction between RIPK1 and RIPK3 lead to formation of the necrosome, a large amyloid-like cytosolic complex that serves as a platform for RIPK3 activation [12]. RIPK3 activation, as measured by the autophosphorylation of human RIPK3 at Ser227 and murine RIPK3 at Thr231 and Ser232, is a key step in the initiation of necroptosis [56–58]. Activated RIPK3 in turn phosphorylates MLKL, which serves as the executor of necroptosis [59]. Phosphorylation of MLKL leads to a conformational change that exposes its N-terminal four-helix bundle domain and leads to the oligomerization of MLKL [60, 61]. Oligomerization leads to a net positive charge of the multi-MLKL complex, and therefore this multi-MLKL complex is recruited to negatively charged membranes through phospholipids, leading to membrane permeabilization and cell lysis [60, 61].

TRIF is activated downstream of TLR3 and TLR4, which are pathogen-recognition receptors of the Toll-like receptor family that initiate immune response through the recognition of pathogen-associated microbial patterns. TLR3 senses viral double-stranded RNA (dsRNA), while TLR4 recognizes lipopolysaccharide (LPS) of Gram-negative bacteria [62]. Analogously to TNFR1, signaling through TLR3 and TLR4 normally leads to proinflammatory and pro-survival responses in the cell due to activation of NF-κB and transcription factor IRF3-mediated production of type I interferons [62]. When cells are sensitized to necroptosis by inhibition of caspase-8, TRIF can activate RIPK1, which in turn promotes RIPK3 activation [54, 55]. However, RIPK1 is dispensable for TLR3-induced necroptosis in mouse fibroblasts, which suggests that RIPK3 can be activated by the binding of TRIF, in addition to RIPK1 [54]. Thus, the involvement of RIPK1 kinase in TLR3/TLR4-mediated RIPK3 activation and necroptosis is cell type-dependent (Fig. 4).

The final RHIM-containing protein encoded by mammals is ZBP1, which was initially described as a sensor of DNA and RNA in the unusual Z-form conformation that is characterized by a left-handed helical arrangement (Z-DNA or Z-RNA) [36]. dsDNA typically adopts the B-conformation, while dsRNA is usually in the A-conformation. ZBP1 has since been shown to elicit cell death and transcriptional responses to DNA viruses via its Z-DNA binding domain [63]. More recent data have shown that ZBP1 can sense Z-form RNA that generated from replication of influenza A viruses (IAV) during infection to trigger RIPK3 activation and necroptosis of macrophages [64]. ZBP1 can also trigger necroptosis in sterile settings in which the ability of RIPK1 scaffold function to suppress necroptosis is disrupted as described above [35]. The ligand that trigger ZBP1 activation in this setting was revealed recently by two independent groups. They demonstrated that de-repressed endogenous retroviral RNA derived from dsRNAs in cells may act as a ligand to prime ZBP1 to mediate necroptosis [65, 66]. Endogenous retroviruses (ERVs) are retrovirus-like elements with long repeats that comprise 5%–8% of the genome in humans [67]. The ERV-derived dsRNA could therefore instruct the assembly of cell death signaling scaffolds, which facilitates RIPK3 activation via RHIM-induced interaction of RIPK3 and ZBP1 [65, 66].

## Role of RIPK1/3 and necroptosis in aging

### Necroptosis in inflammaging

Inflammaging is a hallmark of normal aging, which is closely associated with the development of aging-related diseases and morbidity in the elderly [68]. It has been shown that DAMPs are accumulated with age, which activate the immune system, leading to chronic inflammation, after being released into extracellular environment [69, 70]. Necroptosis is a well-established pathway that promotes the release of DAMPs and triggers inflammation. Therefore, necroptosis may also play a role in aging-related chronic inflammation [71].

Visceral fat tissue (VAT) is an inflammatory organ, which is associated with the greatest inflammatory cytokine production [72, 73]. Activation of necroptosis, as determined by the increase of p-RIPK3 and p-MLKL as well as total protein levels of RIPK1 and MLKL, has been found in epididymal white adipose (eWAT, a visceral fat depot) of old mice [74]. Increased necroptosis in old mice is associated with the production of proinflammatory cytokines and chemokines [74]. Interestingly, calorie restriction (CR) has been shown to inhibit chronic inflammation and retard aging by inhibiting necroptosis pathway and reducing the expression of proinflammatory cytokines in eWAT of old mice [74, 75]. Thus, inhibition of necroptosis acts as a mechanism of action for CR-based modulation of aging. Necroptosis is also associated with longevity in mice. In Ames dwarf mice with lifespan extension, necroptosis and inflammation are both inhibited in eWAT compared with age-matched control mice [71]. In *Sod1*^−/−^ mice with lifespan reduction, necroptosis, and inflammation are both increased in eWAT compared with age-matched control mice [71]. Thus, aging-related increase in necroptosis cause chronic inflammation, which is associated with the length of lifespan, at least in experimental mouse models.

### Necroptosis in brain aging

Aging-related chronic inflammation that present in the central nervous system (CNS) is called neuroinflammation, which plays an important role in the development of aging-related neurodegenerative diseases and aging-related cognitive impairment [76]. The activation of microglia and astrocyte in CNS are important markers of neuroinflammation. The increasing of necroptosis in aging brain has been shown to contribute to the progression of neuroinflammation by releasing DAMPs that can activate microglia and astrocyte [77, 78]. The levels of p-MLKL and oligomerized-MLKL, as well as the expression of RIPK1, RIPK3, and MLKL have been found increased in the neurons of cortex layer V and the CA3 region of the hippocampus in old mice, which triggers the activation of microglia and astrocyte in these regions, leading to the expression of proinflammatory cytokines IL6 and IL1β [79]. Knockout of MLKL or pharmacological inhibition of RIPK1 kinase reduces neuroinflammation by blocking necroptosis of neurons in these regions [79]. Aging-related necroptosis in neurons of hippocampus also contributes to the damage of axonal integrity and function, and adversely impacts synaptic function as well as hippocampal function and memory [80]. Thus, necroptosis plays an important role in driving neuroinflammation and cognitive impairment in aging brain.

### Necroptosis in liver aging

Necroptosis has been shown to contribute to chronic inflammation in aging liver. The levels of necroptosis hallmarks, including p-MLKL, p-RIPK3, and p-RIPK1, are increased in the liver of aged mice [81]. Along with the increase of necroptosis, the markers of M1 proinflammatory macrophages, and that of liver fibrosis are also elevated in aging liver [82]. Notably, pharmacological inhibition of RIPK1 kinase ameliorates M1 macrophages infiltration and liver fibrosis by blocking necroptosis [81]. In a SOD1-deficient mouse model of accelerated aging, necroptosis, along with inflammation and fibrosis are all increased in the liver, which can be reversed by RIPK1 kinase inhibitor [83]. Thus, aging-related increasing of necroptosis plays an important role in the development of chronic inflammation and fibrosis of aging liver.

### Necroptosis in testis aging

Necroptosis has been found to play an important role in normal aging of mouse male reproductive system. RIPK3 or MLKL-deficient male mice show a younger reproductive system compared with age-matched control mice [84]. It is known that seminal vesicles become enlarged as male mice get old, presumably due to secondary growth in response to declining of testis functions [85]. Knockout of RIPK3 or MLKL substantially inhibits aging-related enlargement of seminal vesicles, and prevents the loss of cells in seminiferous tubules in aged testis and the reduction of sperm counts in epididymis of aged mice [84]. RIPK3 or MLKL-deficient male mice also have a significant higher level of testosterone compared with the age-matched control mice [84]. In addition, RIPK3 or MLKL deficiency increases the fertility rates and prolongs the reproductive longevity of aged male mice [84]. In line with this, applying TSZ (TNFα/SM-164/zVAD) treatment, a well-established necroptosis inducer, in young male mice via direct injection into the testis accelerates aging of the reproductive system by initiating necroptosis of seminiferous tubules, suggesting that necroptosis is an adequate condition for aging of the male reproductive system [84]. Mice fed with RIPK1 inhibitor also display delayed aging of the reproductive system by blocking necroptosis, suggesting an upstream of RIPK1 in modulating necroptosis in the aging of male reproductive system [84]. While necroptosis is increased, caspase-8 was found decreased in seminiferous tubules of aged male mice [84]. As discussed above, caspase-8 is a well-established suppressor of necroptosis, thus the reduction of caspase-8 may unleash necroptosis in seminiferous tubules of aged male mice.

### Necroptosis in cochlea aging

Aging-related hearing loss is characterized by an irreversible hearing impairment, which results from the cumulative effects of aging on the auditory system [86]. Aged mice exhibited a significant loss of hearing, number of hair cells, neuronal fibers, and synaptic ribbons compared to young mice. Aged mice also showed significant decrease in cochlear blood flow, and exhibited increase in gene expression of proinflammatory cytokines, including TNFα, IL1β, and IL6 [87, 88]. Interestingly, Necroptosis hallmarks, including RIPK1, RIPK3, and MLKL, were found increased in sensorineural tissues of aging cochlea, which may contribute to enhanced synthesis of proinflammatory cytokines in aging cochlea [88].

## Role of RIPK1/3 and necroptosis in aging-related diseases

### Necroptosis in neurodegenerative diseases

AD is an aging-related neurodegenerative disease, which is characterized by cognitive decline and by the presence of neuronal death, the accumulation of tau tangles, amyloid-β (Aβ) plaques and marked neuroinflammation [89]. Necroptosis has been found activated in postmortem human AD brains, which is positively correlated with Braak stage [90]. Lowering necroptosis activation can reduce neuronal death in mouse model of AD [90]. In granulovacuolar degeneration (GVD) lesions, the presence of necrosome is associated with neurons loss [91]. Moreover, Aβ-mediated microglia activation and TNFα production in AD contributes to necroptosis activation in neurons [92]. In addition to necroptosis activation in AD, RIPK1 is highly expressed in microglial cells in human AD brains, which promotes microglia activation and neuroinflammation, including TNFα and IL1β [93]. Pharmacological or genetic inhibition of RIPK1 kinase reduces neuroinflammation, amyloid burden, and memory deficits in amyloid precursor protein (APP)/presenilin 1 (PS1) transgenic mouse model of AD [93]. A unique subtype of microglia that revealed by single cell RNA sequencing in AD brains, which is called disease associated microglia (DAM), has been recently implicated in mediating the pathogenesis of AD [94]. RIPK1 has been shown to mediate a critical checkpoint in the transition to the DAM state by regulating microglial expression of CH25H and CST7 [93]. Taken together, these studies suggest that targeting RIPK1/3 and necroptosis may be a new therapeutic strategy for AD treatment.

Parkinson’s disease (PD) is considered the second most common aging-related ND, which is characterized by the loss of dopaminergic neurons in striatum and substantia nigra and accumulation of modified α-synuclein in the degenerating neurons termed as Lewy bodies [95]. RIPK1, RIPK3, and MLKL were found highly increased in brains of 1-methyl-4-phenyl-1,2,3,6-tetrahydropyridine hydrochloride (MPTP)-induced PD mice, which is associated with severe loss of dopaminergic neurons [96, 97]. Pharmacological or genetic inhibition of RIPK3 or MLKL to block necroptosis pathway dramatically ameliorated PD pathology by increasing dopamine levels and rescuing the loss of dopaminergic neurons [96, 97]. Necroptosis activation was also found in postmortem brain tissue from PD patients and 6-hydroxydopamine (6-OHDA)-induced PD mice [98]. Inhibition of necroptosis pathway results in a significant delay of 6-OHDA-dependent axonal degeneration of dopaminergic and cortical neurons. Genetic ablation of RIPK3 or MLKL, as well as pharmacological inhibition of RIPK1 decrease dopaminergic neuron degeneration and improve motor performance [98]. Thus, RIPK1/RIPK3/MLKL-mediated necroptosis is involved in the pathogenesis of PD. Therapy targeting DA neuron necroptosis may be a potential strategy for treating PD.

Amyotrophic lateral sclerosis (ALS) is a progressive aging-related neurodegenerative disease of the motor system that predominantly affects the upper and lower motor neurons [99]. ALS belongs to axonal “dying back” neurodegenerative diseases, as the onset begins with axonal pathology. Axonal degeneration makes a substantial contribution to neurological disability in these patients [100]. In a humanized *in vitro* model of ALS, astrocytes derived from individuals with sporadic ALS or familial ALS, killed human embryonic stem cell-derived motor neurons by promoting necroptosis [101], suggesting the role of necroptosis in motor neuron degeneration in ALS. Loss-of-function mutations in optineurin (OPTN) have been implicated in both familial and sporadic ALS [102]. Loss of OPTN resulted in activation of RIPK1 and necroptosis in the spinal cords of *Optn*^−/−^ mice, which was rescued by genetic and pharmacological inactivation of RIPK1 kinase [102]. *Optn*^−/−^ cells and the spinal cords in *Optn*^−/−^ mice show increased levels of total RIPK1 protein, due to decreased proteasomal degradation of RIPK1 protein. TBK1 haploinsufficiency is a monogenic cause of ALS [103]. *Tbk1*^−/−^ mice show profound RIPK1 activation, resulting in embryonic lethality that is rescued by genetic inactivation of RIPK1 [48]. TBK1 directly inhibits RIPK1 by phosphorylation, loss of TBK1 activity increases susceptibility to RIPK1 activation [48]. In addition, age-dependent reduction of TAK1 expression in human brains was shown to cooperate with haploinsufficiency of TBK1 to promote late-onset ALS-like pathology mediated by decreased RIPK1 inhibition [48]. The expression levels of RIPK1, RIPK3, and MLKL were found increased in the spinal cords of postmortem patients with ALS and *Sod1*^*G93A*^ transgenic mice (a mouse model of a rare form of familial ALS)^102^. Knockout of RIPK3 or oral administration of RIPK1 inhibitor in *Sod1*^*G93A*^ mice blocked axonal myelination defects and motor dysfunction onset in these mice [102]. Taken together, these results suggest an important role of RIPK1/3 and necroptosis in the pathogenesis of ALS.

### Necroptosis in cancer

Cancer is a malignant disease associated with abnormal cell proliferation and cell death. Aging is one of the major risk factors for cancer development. Resistance to apoptosis is a hallmark of many types of cancer cells and a major roadblock to traditional chemotherapy. Therefore, induction of necroptosis could be an alternative strategy for anticancer therapy. However, the expression of RIPK3 and MLKL is reduced in various cancer cells, including breast cancer [104], colorectal cancer [105, 106], acute myeloid leukemia [107], and nonsmall cell lung cancer [108]. The reduced expression of RIPK3 and MLKL is associated with worse prognosis and poor survival in these cancers. In addition, RIPK3 expression is silenced in most cancer cells due to genomic methylation [104, 109]. A necroptosis sensitivity screening revealed that 780 (83%) cell lines among a panel of 941 human cancer cell lines from 28 tissues are fully resistant to necroptosis due to the loss of RIPK3 expression that mediated by oncogenes BRAF and AXL [110]. Thus, escape from necroptosis could be a potential hallmark of cancer. Hypomethylating agents or inhibition of these oncogenes may be applied to rescue RIPK3 expression prior to traditional antitumor treatment.

Unlike RIPK3 and MLKL, RIPK1 expression is positively correlated with cancer development. RIPK1 is increased in glioblastoma [111], nonsmall cell lung cancer [112], melanoma [113], and HCC [114]. Increased expression of RIPK1 confers a worse prognosis by regulating cell proliferation and inflammation [111–114], which suggests that RIPK1 may function as an oncogenic driver in cancers. In line with this, administration of RIPK1 inhibitor significantly suppressed tumor growth and development in colitis-associated cancer [115]. Given most of the tumor cells are independent of RIPK3 nor necroptosis, RIPK1 kinase activity has been shown to play a distinct role in tumor-associated macrophages (TAMs). Upregulation of RIPK1 in TAMs has been shown to contribute to immune tolerance and immunotherapeutic resistance in pancreatic ductal adenocarcinoma (PDAC) by switching TAMs toward an M2-like phenotype in a STAT1-dependent manner [116]. Pharmacological inhibition of RIPK1 induces cytotoxic T cell activation and generates an M1-like phenotype that potentiated immunotherapy [116]. Thus, RIPK1 is a checkpoint kinase governing tumor immunity, targeting RIPK1 maybe a potential therapeutic strategy for cancer treatment.

Necroptosis can modulate tumor microenvironment that inhibit tumor progress and invasion by releasing DAMPs, which elicits robust cross-priming of antitumor CD8^+^ T cells via NF-κB pathway [117]. DAMPs from necroptotic cancer cells can also promote maturation of dendritic cells, and the production of IFNγ in response to tumor antigen stimulation [118]. Ectopic introduction of necroptotic cells to the tumor microenvironment promotes BATF3^+^CDC1^−^ and CD8^+^ leukocyte-dependent antitumor immunity accompanied by increased tumor antigen loaded by tumor-associated antigen-presenting cells [119]. In addition to its tumor-suppressive function, necroptosis has been reported to promote tumorigenesis and metastasis. RIPK3 was found highly expressed in pancreatic ductal adenocarcinoma (PDA), deletion of RIPK3 protects against oncogenic progression of PDA, which partially depends on necroptosis-induced expression of CXCL1 that promotes macrophage-induced adaptive immune suppression [120]. RIPK3 and MLKL were also found increased in human pancreatic cancer tissues compared with normal pancreas, which promote pancreatic cancer cell migration and invasion [121]. In leukemic cells isolated from AML patients, RIPK1 and RIPK3 are activated to maintain the undifferentiated state [122]. In addition, the pro-tumoral effects of necroptosis can be also driven by nontumoral cells. For example, necroptosis of endothelial cells promotes tumor cell extravasation and metastasis [123]. Therefore, necroptosis can act via an antitumorigenic or pro-tumorigenic mechanism in a context and cell type-dependent manner. Induction of necroptosis may become a critical strategy against apoptosis-resistant tumors, but its progressive role in cancer should be considered.

### Necroptosis in osteoarthritis

Osteoarthritis (OA) is an aging-related, chronic degenerative joint disorder characterized by pain, cartilage degeneration, and joint dysfunction [124]. Loss of chondrocyte cellularity within the articular cartilage is one of the prominent events that contribute to its degradation. Activation of inflammation-related signaling pathways and chondrocyte death have been shown to play pivotal roles in OA [125]. It has been shown that inhibiting RIPK1 by necrostatin-1s attenuates OA *in vivo* and *in vitro* by blocking RIPK1-HMGB1-TLR4 pathway and apoptosis in a trauma-induced mouse model of OA [126]. RIPK1 is also upregulated in cartilage from both OA patients and experimental OA rat models [127]. Inhibition of RIPK1 protects the rats from trauma-induced cartilage degradation by preventing chondrocyte necroptosis [127]. Necroptosis hallmarks, including RIPK3 and MLKL were found increased in highly degenerated cartilage tissue of OA patients and in human samples collected after intraarticular fracture, which can be attenuated by RIPK1 inhibitor [128, 129]. Taken together, RIPK1/3 and necroptosis are potential therapeutic targets in osteoarthritis treatment.

### Necroptosis in cardiovascular disease

Cardiovascular disease that leads to heart failure and stroke is the leading cause of mortality in the world. Aging is the major risk factor for cardiovascular disease. There is an extensive body of literature identifying myocyte necroptosis as an integrant component in the pathogenesis of myocardial infarction, heart failure, and other cardiovascular disease [130]. It has been shown that pharmacological inhibition of RIPK1 reduces infarct size in models of myocardial ischemia-reperfusion (I/R) injury by preventing necroptosis, reducing inflammatory response and limiting oxidative stress [131, 132]. Cardiac expression of RIPK3 was found upregulated in models of myocardial infarction [133]. Deletion of RIPK3 diminishes inflammatory response, reactive oxygen species (ROS) generation and improves cardiac performance [133]. RIPK3 can also mediate endoplasmic reticulum (ER) stress and CaMKII activation to trigger opening of the mitochondrial permeability transition pore (mPTP) and myocardial necroptosis in models of myocardial ischemia-reperfusion injury [134, 135]. RIPK3 was also reported to be elevated in hypertrophic myocardium tissues from patients and rats that subjected to aortic banding (AB) surgery, which aggravates cardiomyocyte hypertrophy and necroptosis-dependent cardiac injury [136]. Taken together, RIPK1/3 and necroptosis play important roles in the pathophysiology of aging-associated cardiovascular disease, targeting necroptosis pathway may provide therapeutic benefit in the treatment of cardiovascular disease.

### Necroptosis in diabetes mellitus

The prevalence of diabetes mellitus, especially type 2 diabetes (T2D), which is a chronic disease characterized by insulin resistance and insulin deficiency, increases with age. Individuals with diabetes are more likely to develop other aging-related comorbidities, such as AD and cardiovascular disease, suggesting that diabetes may represent a pro-aging state. Islet inflammation is an important etiopathology of diabetes [137]. In mouse model of diabetes, high-fat diet induces ER stress in islets, which activates RIPK3, leading to NF-κB-mediated proinflammatory IL1β expression and β cells dysfunction and loss [138]. Pharmacological inhibition of RIPK3 reduces β cells loss in mouse model of diabetes, suggesting a potential role of necroptosis in T2D pathogenesis [138]. In line with this, deletion of MLKL in mice prevents obesity-induced insulin resistance and glucose intolerance [138]. In addition, pharmacological inhibition of RIPK1 in mice relieves obesity-associated metabolic disturbances [139]. However, MLKL-G316D substitution, which fully ablates MLKL killing activity in human cells was observed exclusively in diabetic patients, suggesting a potential role of impairment of necroptosis pathway in promoting diabetes [140]. In choline-deficient high-fat diet-fed mice and obese humans, RIPK3 is increased in the white adipose tissue (WAT) [141]. However, genetic inactivation of RIPK3 promotes caspase-8-dependent adipocyte apoptosis and WAT inflammation, leading to impaired insulin signaling and glucose intolerance, suggesting a protective role of RIPK3 in diabetes [141]. Thus, RIPK1/3 and necroptosis play an either pathogenic of protective role in diabetes in cell type-dependent manner.

### Necroptosis in atherosclerosis

Atherosclerosis is an aging-related disease, which is characterized by the accumulation of lipid-rich plaques in arterial wall [142]. In the early stage of atherosclerosis, blood-borne monocytes are recruited into the plaques and differentiate into macrophages. Formation of necrotic core in atherosclerosis is associated with necroptosis activation of macrophages, which has been thought to promote plaque rupture and acute atherothrombotic vascular disease [143]. The expression of RIPK3 and MLKL are both increased in human and mouse atherosclerosis [144, 145]. In mouse models of atherosclerosis, such as LDL receptor deficiency or ApoE deficiency, genetic ablation of RIPK3 reduces both advanced atherosclerotic lesions and lesional inflammation [145]. In addition, RIPK3 knockout reduces systematic inflammation and prolongs the lifespan of ApoE-deficient mice [146].

The expression of RIPK1 is also increased in early-stage atherosclerotic lesions of both human atherosclerosis patients and *ApoE*^−/−^ mice [147]. Inhibition of necroptosis in *ApoE*^−/−^ mice with established atherosclerosis by RIPK1 inhibitor reduces atherosclerotic lesion size and markers of plaque instability [144]. However, pharmacological inhibition of RIPK1 in *ApoE*^*SA/SA*^ mice, a model for investigating advanced atherosclerosis, only reduces plaque lesion in early stage, and promotes more atherosclerosis lesions in late stage [148]. Thus, targeting RIPK1/3 and necroptosis is a promising therapeutic strategy for treating atherosclerosis at least in early stage.

### Necroptosis in age-related macular degeneration

PAge-related macular degeneration (AMD) is a degenerative disease of the macula, which is the leading cause of blindness in the elderly [149]. AMD is characterized by extracellular deposits, which is called drusen, formed beneath the retinal pigment epithelium (RPE) at the early stage and geographic atrophy at the late stage [150, 151]. RPE cell damage caused by drusen accumulation plays a key role in AMD pathogenesis [152]. It has been shown that RPE cells undergo necroptosis rather than apoptosis in a mouse model of oxidative stress-induced retinal degeneration, which can be block by RIPK1 inhibitor or silencing of RIPK3 [151, 153]. Drusen-derived dsRNA also promotes necroptosis of RPE, and contributes to inflammation and retinal degeneration, which can be prevented by RIPK3 deficiency [154].

### Necroptosis in nonalcoholic fatty liver disease

Nonalcoholic fatty liver disease (NAFLD) is the most common liver disease worldwide, which is characterized by abnormal accumulation of hepatocellular fat [155]. Aging is a risk factor for NAFLD, which is associated with dysregulation of hepatic lipid metabolism [156]. Hepatocyte apoptosis and necroptosis are cardinal features of NAFLD, which drive the progression of liver inflammation and fibrosis. However, necroptosis may be more important to the pathogenesis of NAFLD based on typical histological features of NAFLD, including necrosis and inflammation [157].

RIPK3 is barely expressed in hepatocytes normally. However, RIPK3 is significantly induced in hepatocytes of patients with NAFLD. Increased RIPK3 level is positively correlated with liver inflammation and fibrosis [158, 159]. In MCD (methionine and choline-deficient) diet-induced mouse model of NAFLD, RIPK3 deficiency reduces liver steatosis, liver inflammation and fibrosis, preventing liver injury [159]. In CDAA (choline-deficient l-amino acid-defined) diet-induced mouse model of NAFLD, RIPK3 deletion ameliorates liver inflammation and fibrosis but increases liver steatosis [158]. However, in HFD (high-fat diet)-induced mouse model of NAFLD, RIPK3 deficiency increases liver inflammation, fibrosis and apoptosis, which exacerbate liver injury, and increase liver weight, liver to body weight ratio and hepatic triglycerides [160, 161]. Thus, the contribution of RIPK3 in NAFLD pathology varies in different experimental models, and the role of RIPK3 in human patients with NAFLD needs to be determined in the future.

RIPK1 is found to be increased in the serum of patients with NAFLD. Treatment of RIPK1 inhibitor in HFD-induced mouse model of NAFLD prevents hepatocyte from necroptosis, subsequently ameliorates liver inflammation and fibrosis. RIPK1 inhibitor also reduces liver steatosis in MLKL-dependent manner in mice with NAFLD. Inhibition of RIPK1-MLKL axis can increase hepatocellular mitochondrial respiratory, followed by decreased hepatocellular triglycerides, thus reversing the progression of NAFLD [162]. Taken together, these results suggest that RIPK1/3 and necroptosis play key roles in the pathogenesis of NAFLD.

### Necroptosis in retinal degeneration

Retinal degeneration is the major causative factor to induce irreversible vision loss even blindness, which is characterized by the progressive loss of photoreceptors leading to retinopathy [163]. Aging is a risk factor for retinopathy, and retinal degeneration also develops in some age-related diseases. In age-related macular degeneration, the separation of neurosensory retina from RPE causes photoreceptors death and retinal degeneration [164]. In age-related neurodegenerative diseases, such as PD and AD, the loss of retinal ganglion cell contributes to the progress of retinal degeneration [165]. Chronic inflammation also plays an important role in driving retinal degeneration in these diseases [166]. With aging, microglia in retina undergoes RIPK1 and RIPK3-dependent necroptosis, which is activated by TLR4 pathway [167]. The necroptosis of microglia promotes the release of various proinflammatory cytokines such as TNFα and CCL2, which induces retinal degeneration [167]. Accordingly, RIPK1 inhibitor blocks microglia necroptosis and microglia-mediated inflammation, preventing retinal degeneration [167].

### Necroptosis in chronic kidney disease

Chronic kidney disease (CKD), characterized by interstitial fibrosis and a gradual loss of kidney function, is a common disease of aging [168–170]. Interstitial fibrosis is caused by the damage of renal tubules and interstitial capillaries as well as consequent replacement of the extracellular matrix [171]. In mouse unilateral ureteral obstruction (UUO) model of CKD, necroptosis is increased, and positively correlated with interstitial fibrosis [172]. RIPK1 inhibitor has been shown to suppress interstitial fibrosis through blocking necroptosis of renal tubule cells and inflammation in mice with UUO [172]. In rat noninflammatory subtotal nephrectomy model of CKD, necroptosis contributes to the progressive loss of renal tubule cells, thereby aggravating renal fibrosis and promoting the progression of CKD, which can be alleviated by RIPK1 inhibitor [173, 174]. Angiotensin II (AngII) has been reported to exert key and adverse effects on progression of renal fibrosis and CKD [170]. Treatment of RIPK1 inhibitor in AngII-induced renal injury and fibrosis mouse model prevents renal tubular epithelial cells from necroptosis and attenuates interstitial lesion, suggesting an important role of RIPK1 kinase in the development of renal fibrosis and CKD in AngII-induced renal injury and fibrosis mouse model [170].

There is an extensive body of literature identifying necroptosis as a key pathogenic mechanism in the progression of renal interstitial fibrosis in experimental models. However, genetic ablation of MLKL does not offer any benefit in UUO mice model [175]. In contrast, RIPK3 deficiency prevents UUO-induced kidney fibrosis, supporting the notion that RIPK1 and RIPK3 regulate more than just MLKL-dependent necroptosis [175]. Indeed, RIPK3 deficiency or catalytically inactive RIPK1 provides greater benefit than MLKL deficiency in several mouse models of inflammation and tissue injury, such as kidney ischemia-reperfusion injury [176]. It has been proposed that RIPK3 loss may have the added effect of suppressing the production of proinflammatory cytokines and chemokines independent of cell death [176]. However, in general, targeting RIPK1 or RIPK3 may be a potential therapeutic strategy for treatment of aging-related renal interstitial fibrosis and CKD at least in experimental models.

### Necroptosis in COVID-19

Coronavirus disease 2019 (COVID-19) is a pandemic disease caused by the severe acute respiratory syndrome coronavirus 2 (SARS-CoV-2) infection [177]. The clinical manifestations of COVID-19 have a broad spectrum ranging from asymptomatic cases to acute respiratory distress syndrome and even death [178]. Aging which causes a poor state of health is the primary risk factor for the development of severe COVID-19 and death [179, 180]. In severe COVID-19, the hyperactivation of immune system and dysregulation of coagulation pathways result in overproduction of proinflammatory cytokines, which is called cytokine storm, development of thrombosis, which causes lung injury, disseminated intravascular coagulation and multiple organ failure [181, 182].

Activation of necroptosis pathway has been found in multiple tissues of severe COVID-19 patients. The hallmark of necroptosis, p-MLKL, has been detected in the lung section of COVID-19 patients and SARS-CoV-2-infected human ACE2 transgenic mice [183]. The protein levels of RIPK1, RIPK3, and MLKL are also elevated in the plasma of both moderate and severe COVID-19 patients [184]. Plasma of severe COVID-19 patients can induce a necroptosis-sensitive neutrophil phenotype, which has also been detected in the biopsies of COVID-19 thrombus and lung [185]. In addition, necroptosis has been found in platelets of COVID-19 patients [186]. Platelets take up SARS-CoV-2 virus and undergo rapid necroptosis to limit viral replication. However, when this response exaggerated, activated platelets induce massive neutrophil necroptosis and the release of neutrophil extracellular traps, which contributes to dysregulated immunity and thrombosis [186].

RIPK1 kinase activation has been found in human COVID-19 lung pathological samples, cultured human lung organoids and lung section of human ACE2 transgenic mice infected by SARS-CoV-2 [187]. Mechanistically, NSP12, the RNA-dependent RNA polymerase of SARS-CoV-2, interacts with RIPK1 and drives its kinase activation, which subsequently increases the expression of RIPK1 kinase-dependent proinflammatory cytokines [187]. Interestingly, the activation of RIPK1 kinase also promotes the expression of host factors, such as ACE2 and EGFR, which facilitates the entry of SARS-CoV-2 virus in host cells [187]. Thus, the activation of RIPK1 kinase not only drives cytokine storm, but also unexpectedly enhances viral survival and replication during SARS-CoV-2 infection. Accordingly, pharmacological inhibition of RIPK1 kinase reduces the replication of SAR-CoV-2 in human lung organoids and increases the survival of human ACE2 transgenic mice infected by SARS-CoV-2 [187]. The important role of RIPK1 kinase in the pathogenesis of COVID-19 has also been revealed by proteome-wide data analysis, which suggests RIPK1 as a promising drug target to rescue multiorgan injury and inflammation in COVID-19 [187]. In conclusion, targeting necroptosis and RIPK1 kinase may be a promising strategy to treat aging-related severe COVID-19 patients.

**Figure 5.**
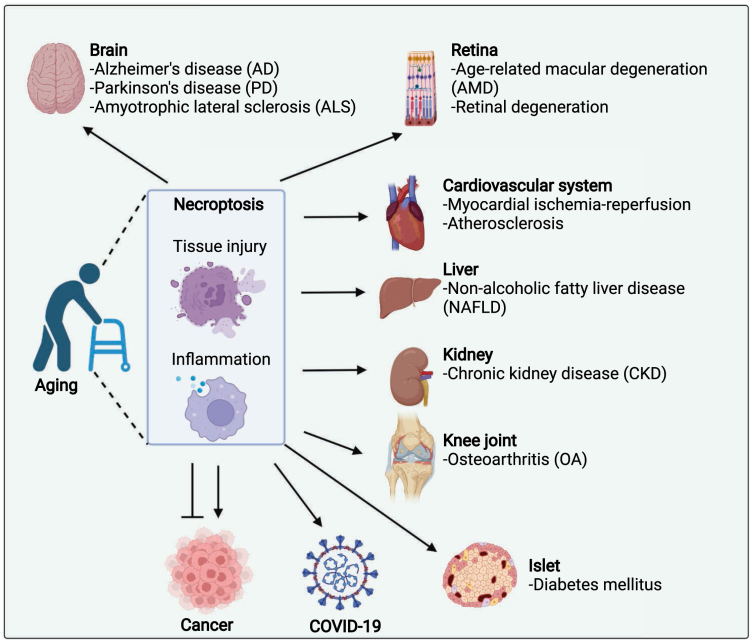
Role of necroptosis in aging-related diseases. Necroptosis has been identified in various aging-related diseases, including neurodegenerative diseases, cancer, cardiovascular disease, atherosclerosis, osteoarthritis, diabetes mellitus, nonalcoholic fatty liver disease, age-related macular degeneration, retinal degeneration, CKD, and COVID-19.

In summary, recent studies demonstrated that RIPK1, RIPK3, and necroptosis play important roles in the progression of a variety of aging-related diseases based on the activation of RIPK1/3 and necroptosis in each of these diseases (Fig. 5). Importantly, blocking RIPK1/3 and necroptosis during these aging-related conditions resolved tissue degeneration and inflammation, which reduced the progression of the disease, suggesting a potential therapeutic strategy for treating these aging-related diseases by targeting RIPK1/3 and necroptosis.

## Concluding remarks

Recent studies show that the increase in necroptosis contributes to chronic inflammation and tissue degeneration that increase with age, and blocking or reducing necroptosis either genetically or pharmaceutically can reduce inflammation and tissue degeneration in both normal aging and aging-related diseases. RIPK1 and RIPK3 are recognized as potential therapeutic targets for many diseases, given their broad linkage to the pathogenesis of inflammatory diseases, aging-related diseases, and even the normal aging process. Greater understanding of these biochemical functions of RIPK1/3 in normal aging and aging-related disease models will certainly be instrumental for the development of valuable antiaging therapeutic strategies. With ongoing advances of increasing number of RIPK1 inhibitors in human clinical studies for the treatment of many different human inflammatory and degenerative diseases, we expect that studies of RIPK1 in normal aging and aging-related diseases will continue to provide helpful guidance and new exciting directions.

Inhibiting RIPK1/3 and necroptosis has significant effects in antiaging and in attenuating aging-related diseases and displays promising perspective in aging-related clinical applications. However, distinct molecular mechanisms through which aging promotes the activation of RIPK1/3 and necroptosis are still poorly understood and remain to be investigated in great detail. RIPK1 has dual functions which can promote necroptosis mediated by its kinase activity, or prevent necroptosis mediated by its scaffold function. The kinase activity of RIPK1 has been demonstrated to play an important role in aging and aging-related diseases, it remains unknown whether RIPK1 scaffold function involves in the regulation of aging and aging-related diseases. Given the important role of ZBP1 and TRIF in driving necroptosis in addition to RIPK1, the potential role of ZBP1 and TRIF-dependent necroptosis in aging and aging-related diseases may represent a new exciting direction. Better understanding of these mechanisms will greatly facilitate the development of novel and efficient strategies for the prevention and treatment of aging and aging-related diseases by modulating RIPK1/3 and necroptosis.
